# Classic Chinese Acupuncture versus Different Types of Control Groups for the Treatment of Chronic Pain: Review of Randomized Controlled Trials (2000–2018)

**DOI:** 10.1155/2019/6283912

**Published:** 2019-12-04

**Authors:** Yan-Jiao Chen, Gabriel Shimizu Bassi, Yong-Qing Yang

**Affiliations:** Shanghai Research Institute of Acupuncture & Meridian, Shanghai University of Traditional Chinese Medicine, 650 South Wanping Road, Shanghai 200030, China

## Abstract

**Objective:**

To review the effectiveness of classic Chinese acupuncture in the treatment of chronic pain by comparing treatment groups with different types of control groups in accordance with the newly published guidelines for systematic reviews.

**Methods:**

We searched EMBASE, PubMed, and the Cochrane Central Register of Controlled Trials databases from 2000 to 2018. We included randomized controlled trials that included acupuncture as the sole treatment or as an adjunctive treatment for chronic pain. The outcome was pain intensity measured by the visual analogue scale (VAS), Western Ontario and McMaster Universities Osteoarthritis Index (WOMAC) pain subscale, 11-point numeric rating scale (NRS), and other tools. Two researchers conducted the study selection, data extraction, and quality assessment processes independently. Disagreements were solved by discussion and reanalysis of the data. The quality of all included studies was evaluated using the CBNG (the Cochrane Back and Neck Group) and the STRICTA (Standards for Reporting Interventions in Controlled Trials of Acupuncture) checklists.

**Results:**

Sixty-one studies were fully analyzed and ranked based on the newest STRICTA and CBNG standards. We found good evidence that receiving acupuncture is better than not receiving treatment or being placed on a waiting list and reasonable evidence that it is better than conventional or usual care. Limited evidence was found regarding placebo treatments that involve the expectation of needling (real or fake).

**Conclusion:**

Sham acupuncture may not be appropriate as a control intervention for assessing the effectiveness of acupuncture. Acupuncture effectiveness in controlling chronic pain is still limited due to the low quality of the studies published.

## 1. Introduction

At least one-third of the world's adult population experiences some types of physical pain conditions [1]. Chronic pain (CP) is a continuous health problem that persists or recurs for 3 or more months [2] and is a common complaint in approximately 20% of the European population, 11% to 40% of the North American population [3], and approximately 35% of the Chinese population [4]. CP has a direct impact on daily life activities [5, 6], mental health [5, 7], employment [5, 8], and economic well-being [6], and it is one of the most common compelling reasons that adults seek medical attention [9]. In addition, it is estimated that CP-related conditions caused annual economic losses that are estimated to be more than €200 billion in Europe and U$150 billion in the USA [3].

Acupuncture is a therapeutic technique in which needles are inserted into specific points of the body (acupoints) [10, 11]. Clinical studies have showed that acupuncture therapy can improve CP-related conditions, such as neck and shoulder pain [12–14], osteoarthritis (OA) [15, 16], and knee [17] and low back pain (LBP) [18]. However, other studies have indicated that acupuncture therapy is ineffective [19, 20], and systematic reviews have reported inconclusive results [21–23]. These efficacy evaluations were performed by comparing an acupuncture treatment group with different control groups, which were not differentiated. There is a growing awareness that acupuncture control groups in current clinical trials are not properly designed, for example, sham acupuncture, which may be effective [24, 25]. The types of control groups included in acupuncture clinical trials might affect the conclusion of the trials [26]. Additionally, most of these reviews were based on studies published before the 2000s, and since then, several methodology guidelines for systematic reviews have been published and updated. For example, the Standards for Reporting Interventions in Controlled Trials of Acupuncture (STRICTA) was released in 2002 and was updated in 2010 [27, 28], and the Cochrane Back and Neck Group (previously Cochrane Back Review Group) was updated in 2003, 2009, and 2015 [29]. Therefore, a comprehensive review based on the newest standards for acupuncture safety and effectiveness is needed.

In the present study, we performed a review of randomized controlled trials (RCTs) published between 2000 and 2018 regarding the effect of acupuncture therapy on the relief of CP-related conditions. The selected studies were ranked based on the newest STRICTA and CBNG standards, the effectiveness of acupuncture was assessed by comparing acupuncture treatment groups with different types of control groups, and the methodologies, such as the treatment and follow-up duration, needling method, acupoints selection, depth of needling, and number of sessions, were screened and compared.

## 2. Materials and Methods

### 2.1. Search Methods and Data Acquisition

Trials published between 2000 and 2018 were first extracted from EMBASE, PubMed, and the Cochrane Central Register of Controlled Trials databases. Trials conducted by Chinese groups (inside or outside of China) and trials from Chinese databases, such as the China National Knowledge Infrastructure (CNKI), the Traditional Medical Literature Analysis and Retrieval System (TCMLARS), and the Wanfang database, were not considered for the present study. We avoided studies conducted by Chinese groups due to the specific acupuncture schedule that has been developed with long-term clinical experience that is usually used in the implementation of chronic pain randomized controlled trials conducted by Chinese doctors [30]. Our search strategy is shown in the Supplementary Material ([Supplementary-material supplementary-material-1]).

First, the titles and abstracts of all the articles were screened and were assessed by two reviewers (CYJ and GSB). If it was included in the review, the study was then fully analyzed. Any disagreements were solved by discussion and reanalysis of the data.

### 2.2. Selection Criteria

#### 2.2.1. Types of Studies

*(1) Inclusion Criteria*. (1) Studies on chronic pain (pain lasting more than 3 months prior to the inclusion in the study); (2) randomized controlled trials (parallel and/or crossover studies); (3) full articles; (4) studies published in the English language; and (5) studies that included pain as the primary outcome.

*(2) Exclusion Criteria*. (1) Studies in nonhuman animals; (2) nonrandomized or quasirandomized trials; and (3) case reports, abstracts, series, conference reports, comments, and letters.

#### 2.2.2. Types of Participants

*(1) Inclusion Criteria*. (1) Patients suffering from chronic musculoskeletal pain, such as low back pain (LBP), knee or hip arthritis (OA), pelvic or neck pain, shoulder pain and subacromial impingement, epicondylitis, tension-type headaches, myofascial pain, and fibromyalgia; and (2) patients ≥18 years old.

*(2) Exclusion Criteria*. (1) Healthy volunteers; (2) pregnant individuals or those undergoing menopause; (3) individuals with cancer-related pain; (4) individuals with central neurological conditions; and (5) individuals with menstruation-related pain.

#### 2.2.3. Types of Interventions

*(1) Inclusion Criteria*. (1) Manual acupuncture (intervention in which needles are manually inserted into acupoints to reach the subcutaneous tissue [28]); and (2) “Deqi” sensation (numbness, aching, spreading, radiating, dull, heavy, pressure, relieving, and/or electrical feelings from the deep stimulation of the acupoint) was optional.

*(2) Exclusion Criteria*. (1) Other therapies (nonclassic) in addition to or similar to the needle acupuncture, such as auricular, tongue, microsystems, intradermal or laser treatments, acupressure, apipuncture, scalp treatments, facial treatments, or transcutaneous electrical nerve stimulation (TENS), were not considered; and (2) mixed interventions (e.g., manual acupuncture + electroacupuncture) if manual acupuncture was not considered the main intervention.

#### 2.2.4. Types of Controls

*(1) Inclusion Criteria*. (1) No treatment or a waiting list; (2) usual care (including medicine therapy); (3) physiotherapy; (4) relaxation; (5) self-educational programs; (6) manipulation; (7) superficial acupuncture; (8) nonpenetrating needles; (9) insertion simulation at nonacupoints; and (10) application of placebo TENS or laser.

#### 2.2.5. Types of Outcomes

*(1) Inclusion Criteria*. (1) Pain intensity as the main outcome and (2) pain intensity measured immediately after the treatment or up to 1 week after the end of the treatment.

*(2) Exclusion Criteria*. (1) Pain intensity only measured more than 1 week after the end of the treatment and (2) the absence of a complete descriptive data analysis.

### 2.3. Data Analysis

We based our analysis on the following scales (in order of importance): (1) Visual Analogue Scale (VAS); (2) Numerical Rating Scale (NRS); (3) McGill Pain Questionnaire (MPQ); (4) Western Ontario and McMaster Universities Osteoarthritis (WOMAC); (5) Short-Form Health Survey (SF-36); (6) Shoulder Pain and Disability (SPADI); (7) Knee Society Score (KSS); (8) Schmerz empfindung skala (SES); (9) Von Korff Chronic Pain Grade Scale (CPGS); (10) Chronic Prostatitis Symptom Index (CPSI); (11) the Northwick Park Questionnaire (NPQ); (12) Brief Pain Inventory (BPI); and (13) Symptom Bothersomeness Score (SBS). This review is reported in accordance with PRISMA (Preferred Reporting Items for Systematic Reviews and Meta-Analyses) and STRICTA. For crossover trials, we considered data only from the first period due to the carry-out effect.

### 2.3.1. Bias Risk and Quality Assessment

Two reviewers (CYJ and GSB) independently assessed the risk of bias for each study. The quality of each trial was composed of sixteen “yes” (1 point), “no,” or “not informed” (0 points) items based on the 2015 Cochrane Back Review Group [29] and 2010 STRICTA guidelines (Table 1). Low-quality studies were considered those for which the total score was equal to or lower than 8 points. Medium-quality studies were considered those for which the total score ranged between 9 and 13 points. A study was considered of high quality if the total score was equal to or higher than 14 points.

## 3. Results

### 3.1. Included Studies

**Figure 1:**
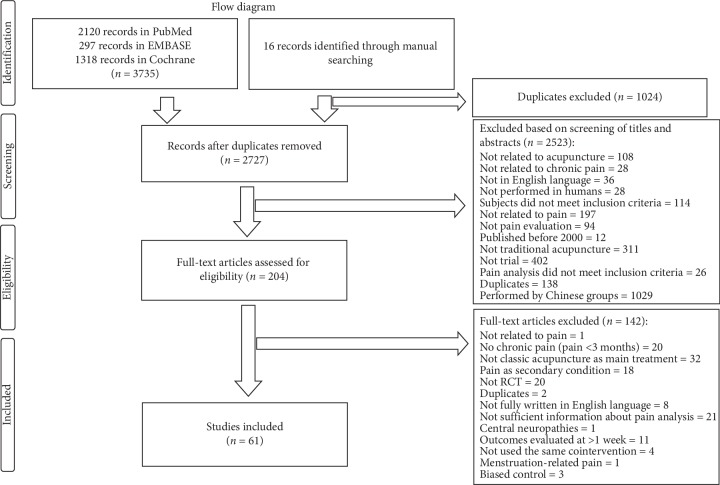
Flow diagram of the trial selection process.

A total of 3735 studies were selected based on our standard search methods, and 16 trials were added by manual search. A total of 1024 duplicates were excluded, and 2523 records were removed based on the title and abstract screening process. A total of 204 full-text articles were assessed for eligibility. Of these, 142 reports were excluded during the selection process (Figure 1). In the present review, 61 studies were used for the final analysis.

### 3.2. Study Characteristics

The 61 selected studies were published between 2000 and 2018. The number of participants in the studies varied from 16 to 3766 (median: 90; IQR: 39 to 227; total 20389). Fourteen studies were conducted in Germany, nine were conducted in the USA, seven were conducted in the UK, seven were conducted in Japan, four were conducted in Spain and Turkey, three were conducted in Iran, two were conducted in Australia, Ireland, and Italy, and one was conducted each in Brazil, Belgium, Canada, Israel, Korea, Malaysia, and Sweden.

#### 3.2.1. Condition Characteristics

Fourteen trials addressed low back pain (6736 patients); eight addressed headaches (4296 patients); seven addressed neck pain (4659 patients); six addressed knee pain (1596 patients) and shoulder pain (1206 patients); four addressed jaw pain (89 patients); three addressed arm-related pain (218 patients) and pelvis and hip pain (221 patients); and one addressed hip and knee pain (712 patients) and back and neck pain (109 patients). In addition, three studies evaluated myofascial pain (114 patients) and five addressed fibromyalgia (433 patients).

#### 3.2.2. Patient Characteristics

The proportion of females ranged from 0% to 100% (median: 66.3%; IQR: 52.27 to 82.675). One study included only males, six included only females, 53 included both sexes, and one did not report sex. The studies included only adult patients (≥18 years old), and the average age varied from 21.7 to 77.44 years (median: 47.7; IQR: 41 to 58.575). Pain duration ranged from 3 months to 21.6 years, and baseline pain intensity ranged from 26.8 to 77.9 (median: 65.05; IQR: 60.975 to 67.275) for the VAS (21 studies) and from 4 to 9.2 (median: 7.275; IQR: 5.8525 to 8.045) for the VAS (0–10) (16 studies). Other pain or pain-related scales that were used included the SF-36 (6 studies), WOMAC (5 studies), SPADI, NRS, and MPQ (2 studies each), and SES, KSS, CPSI, CPGS, BPI, NPQ, and SBS (1 study each). No significant difference was reported in the baseline parameters among groups in all included studies, except in one [31].

#### 3.2.3. Intervention Characteristics

The number of treatment sessions ranged from 1 to 30 (median: 9; IQR: 6 to 13.5); the treatment frequencies reported ranged from 1 (0.67) to 5 times/week (median: 1.33; IQR: 1 to 2). The total treatment period ranged from 1 to 26 weeks (median: 6; IQR: 4 to 9). The mean duration for each acupuncture session ranged from 10 to 50 minutes (median: 20; IQR: 20 to 30); six studies did not report the length of the sessions, and in 4 studies, the sessions involved removing the needle immediately after its insertion. The total duration of the follow-up period ranged from 2 to 84 weeks (median: 12; IQR: 5 to 19). Some of the trials did not provide clear information about the number of acupoints used, especially for individualized interventions in which the number of acupoints varied at the discretion of the therapist. Estimations were based on the report descriptions from the trials. The number of acupoints ranged from 1 to 33 (median 9; IQR 6 to 11.625).

#### 3.2.4. Control Characteristics

Placebo procedures using sham acupuncture were identified as either penetrating or nonpenetrating. Penetrating sham acupuncture involved puncturing sham locations (e.g., needles inserted in areas distal to real acupoints or in acupoints not related to the painful condition) or sham insertions (e.g., superficial insertion into real or nonacupoints, or the use of blunt, nonpenetrating, needles). Eight studies used superficial needling as a control intervention, of which 5 punctured nonacupoints [17,32–34] and 3 used superficial needling at real acupoints [35–37]. Twelve studies performed punctured sham acupoints at the same depth as the real intervention [38–49]. Eighteen studies used nonpenetrating needles, of which 14 studies applied them at the same acupoints of the treatment group [31,45,50–61] and 4 used nonacupoints [39,62–64]. Thirty-three studies used other types of control interventions. Sixteen adopted conventional care as a control intervention (e.g., physiotherapy, self-educational programs, relaxation, manipulation, and usual care) [32, 48, 51, 60, 65–76]. Eight trials reported using pharmacological interventions (e.g., botulinum toxin A, metoprolol, lidocaine, dibucaine hydrochloride, fluoxetine, ibuprofen, Celebrex/ Vioxx/paracetamol, sodium valproate, or diclofenac) [40, 75, 77–82]. Four studies used placebo TENS [83–86], and one applied a laser or sham laser at acupoints as the control intervention [87]. Two studies adopted a waiting list control group [17, 33]. In three studies, the usual medical care was not detailed [88–90], and one did not provide any treatment to the control group [87]. The overall information on each painful condition is shown in Table 2.

### 3.3. Risk of Bias and Methodological Design

**Figure 2:**
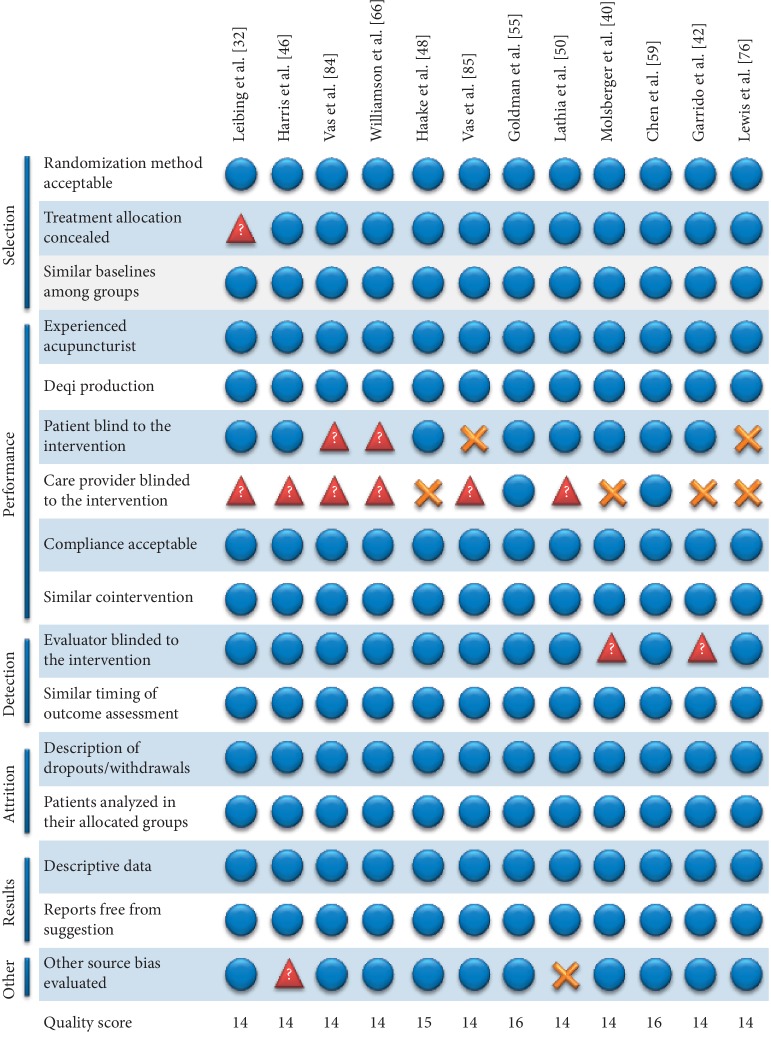
Methodological characteristics of the twelve best-scored trials. 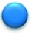 stands for YES, 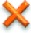 stands for NO, and 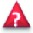
stands for UNKNOWN.

The quality scores of all studies ranged from 7 to 16 (median 12; IQR 11 to 13). Four studies scored 8 or fewer points [35, 70, 88, 90]. Forty-five studies were considered of medium quality and scored between 9 and 13 points. The remaining 12 trials achieved ≥14 points and were considered of high quality [32, 40, 42, 46, 48, 50, 55, 59, 66, 76, 84, 85]. All low-quality trials associated acupuncture with positive results. In the medium-quality group, seven studies reported a neutral effect of acupuncture, two reported a negative effect compared with the control group, and the remaining 36 reported positive effects. In the high-quality group, three trials reported neutral effects, two reported negative outcomes, and seven reported positive results compared to the control (Table 3). Fifty studies reported that acupuncture was performed by an experienced professional. Twenty-three reports did not state whether “Deqi” was induced during the procedures, and 1 trial [73] reported that “Deqi” was induced in most patients in the treatment group. Fifty-five trials specified the method of patient randomization, while six studies failed to provide a detailed description of the randomization process. Twenty-six studies reported the use of allocation concealment (opaque sealed envelopes or a central call service), and 35 studies did not report this method clearly. Concerning bias related to blinding, in 23 studies, the patients were not blinded to the intervention, 55 did not provide a sufficient amount of information about the blinding of care providers, and 21 reports did not blind the evaluators to the outcomes or did not provide a sufficient amount of information. Thirty-four studies reported reasons for patient dropouts and withdrawals. Three reports did not use the same timing among groups to evaluate treatment outcomes. In three studies, patients did not remain in their original randomization groups, and in 3 trials, this issue was not mentioned. Twelve studies did not provide a sufficient amount of information on the presence of other potential risk biases other than those in the initial screening process. Three of these studies showed inconsistencies in the baseline parameters. Two trials showed significantly higher baselines for the control group: one on the Fibromyalgia Impact Questionnaire Score [61] and the other on the duration of headaches and in the Mental Component Score [74]. One study showed a higher baseline level for the acupuncture group regarding the score for back function and pain duration [90]. Five studies did not mention baseline parameters [17, 31, 35, 36, 51]. Two articles did not fully report whether suggestion bias was avoided. Ten studies did not show complete descriptive data about the pain outcomes. One study failed to report a proper baseline for the pain scales (WOMAC and VAS) [31]. The overall checklist reporting for the eligibility of the 12 high-quality studies is shown in Figure 2.

### 3.4. Outcomes of the Acupuncture Groups Compared with Those of the Control Groups

#### 3.4.1. Acupuncture Groups versus No Treatment or Waiting List Control Groups

Acupuncture therapy was compared with no treatment in three studies, of which two used waiting lists [17, 33] and one used no treatment as an intervention [87]. The WOMAC pain scale was used in two studies related to knee OA [17] and knee pain [87], and the VAS was used in one study related to LBP [33]. The acupuncture group showed modest improvements in knee pain after 12 weeks of treatment and along a 1-year follow-up [87]. On the other hand, individuals with knee OA showed improved WOMAC scores after 8 weeks of acupuncture treatment compared to the waiting list group [17]. After eight weeks of acupuncture therapy, the VAS and SF-36 scores were improved in the acupuncture group compared to the waiting list group [33].

#### 3.4.2. Acupuncture versus Pharmacological Interventions

Seven studies used the VAS and one used the SES [78] to report the effects of acupuncture versus a pharmacological intervention. The pharmacological interventions included Celebrex, Vioxx, and/or paracetamol [75], fluoxetine [81], ibuprofen [82], metoprolol [78], sodium diclofenac [40], or sodium valproate [77], or local injections of botulin toxin [77], lidocaine [79], or dibucaine [80]. Five trials showed improved pain outcomes after 4 weeks of acupuncture treatment in individuals with carpal tunnel syndrome (vs. 10 days of ibuprofen) [82], fibromyalgia (vs. 8 weeks of fluoxetine) [81], and LBP (vs. 4 weeks of dibucaine) [80]; after 6 weeks of acupuncture treatment in individuals with chronic shoulder pain (vs. daily diclofenac) [40]; and after 8 weeks of acupuncture treatment in individuals with migraine (vs. 12 weeks metoprolol) [78]. Two studies reported no difference between the effects of acupuncture and lidocaine injections on trigger points (myofascial pain) after 3 weeks and along 14 days of follow-up [79], or 9 weeks of analgesic cocktail (Celebrex, Vioxx, and/or paracetamol) on LBP [75]. One study reported improved pain outcomes after botulin A injections for headaches (trigger points) (8 weeks) or the administration of sodium valproate (12 weeks) compared to real acupuncture at 1 month after the end of the treatment. However, acupuncture was superior to medicine at the 2- and 3-months of follow-ups [77].

#### 3.4.3. Acupuncture versus Usual Care

Three studies showed the effects of acupuncture versus usual care [51, 72, 73]. Three trials favored acupuncture, in which SF-36 pain scores were improved at 3-month and at 12- and 24-month follow-ups after treatment for headaches [72] or after 10 interventions for the treatment of LBP [73]. In addition, one trial showed a significant reduction in the SBS (0–10) score after 7 weeks of acupuncture treatment and at 8-, 26-, and 56-week follow-ups compared to usual care [51]. However, it is important to note that although acupuncture improved LBP, there were no significant differences among the standardized, individualized, and simulated acupuncture groups [51].

#### 3.4.4. Acupuncture plus Medical/Usual Care versus Medical/Usual Care Alone

Five trials used acupuncture as an adjunct therapy to usual medical care for 4 different conditions: knee/hip OA [88], neck pain [71, 89], LBP [90], and headache [74]. Three studies from the same author reported improved pain control in patients under acupuncture/medical care treatment for knee/hip OA, neck pain, or LBP [88–90] in the patients under medical care alone. For knee/hip OA, acupuncture plus usual care showed greater improvement in the WOMAC pain score after 3 months of treatment and up to 6 months in the follow-up period [88]. Similar outcomes were observed when using the SF-36 scale for the evaluation of neck pain, LBP, and headaches [74, 89, 90]. In these studies, individuals with neck pain and headaches but not those with LBP in the acupuncture plus usual care group showed improved SF-36 scores at 3- and 6-month follow-ups than the usual care group. A similar effect was observed when the NPQ scale was used for assessing neck pain and associated disabilities. The addition of acupuncture to usual care showed a better prognosis on NPQ at the end of the intervention (3 months) and at the 12-month follow-up than usual care alone [71].

#### 3.4.5. Acupuncture versus Physiotherapy

Three trials compared the effectiveness of acupuncture against physiotherapy in individuals with three conditions: LBP [48], tension-type headache [65], and knee OA [66]. For LBP, acupuncture showed superior improvement in the CPGS score that lasted up to 6 months after 5 to 7 weeks of acupuncture treatment [48]. For tension-type headaches, 10 to 12 weeks of acupuncture or physical training significantly decreased the VAS pain score immediately after the end of the interventions and at the 6-month follow-up, but this difference was not significant between the groups [65]. For knee OA, both acupuncture and physiotherapy decreased the WOMAC and VAS pain scores at 7 and 12 weeks after the treatment compared to the baseline, but no significant difference was shown between groups [66].

#### 3.4.6. Acupuncture plus Physiotherapy versus Physiotherapy Alone

Three studies reported the effects of acupuncture as an adjunct therapy to physiotherapy on LBP control [32, 67, 68]. In one trial, more favourable SF-36 scores at the 12-week follow-up were reported by patients under acupuncture and physiotherapy than by patients treated only with physiotherapy [67]. Two studies showed lower VAS pain intensity after 12 or 4 weeks of acupuncture in combination with physiotherapy treatment compared to physiotherapy alone [32] and physiotherapy plus diclofenac [68], respectively.

#### 3.4.7. Acupuncture versus Relaxation

Two trials compared the effectiveness of acupuncture and body relaxation in treating chronic headaches and whiplash-associated conditions [65, 69]. One study showed that 8 to 10 weekly sessions of relaxation were more effective in reducing headache-related pain than 10 to 12 weeks of acupuncture treatment [65]. However, this effect was not significant at the follow-ups on weeks 12 and 24. The other trial showed that listening to a CD with relaxing music (20 minutes) or a brief acupuncture treatment (one session) reduced whiplash-associated pain conditions [69], but no difference was found between groups at any time point (immediately and at the 3- and 6-month follow-ups). Notably, the duration and number of days without headaches were significantly higher for the relaxation group [69].

#### 3.4.8. Acupuncture versus Self-Educational/Exercise Programs

Five trials evaluated the effects of acupuncture versus self-educational programs on hip OA, knee OA, neck, and subacromial pain. Two studies showed improved WOMAC pain scores for individuals with hip OA [70] and knee OA [60] after 14 and 26 weeks and at the 8- and 26-week follow-ups, respectively, for the real acupuncture group. Neck pain was evaluated after 12 acupuncture sessions compared to 20 one-to-one Alexander Technique lessons [71]. Although both interventions significantly improved the NPQ score for neck pain and associated disability, there was no difference in the NPQ score between the groups at the 12-month follow-up. One trial showed that acupuncture is not superior to self-exercise in improving the SPADI score after 6 weeks of treatment and at the 6- and 12-month follow-ups [76]. One study showed greater improvement in the VAS scores for patients with knee OA under a self-exercise regimen at the 6-week follow-up than for the acupuncture group [66].

#### 3.4.9. Acupuncture versus Manipulation

One trial reported the effects of acupuncture versus chiropractic spinal manipulation on chronic neck pain [75]. In this study, the manipulation group showed improved outcomes according to the SF-36 and VAS scores for back pain immediately after the end of the treatment. However, the acupuncture group showed greater improvement after 2, 5, and 9 weeks of treatment in the VAS score for neck pain.

#### 3.4.10. Acupuncture versus Superficial Needling

Eight studies used superficial or minimal needling as sham controls: three trials used superficial needling at acupoints (SNA), three studies used superficial needling at nonacupoints (SNNA), and two trials used superficial needling at nonacupoints as an adjunct therapy to other interventions.

In studies using SNA as a control treatment, one study reported significant effects of real acupuncture on the MPQ score only at the 3-month follow-up, but not during 6 weeks of treatment in individuals with lumbar myofascial pain [36]. One study evaluated shoulder myofascial pain and reported that the real acupuncture group showed better MPQ scores at the end of 4 weeks of treatment and at the 4- and 12-week follow-ups [35]. One study evaluated LBP and reported that both interventions (superficial vs. real acupuncture) reduced VAS pain intensity after 4 weeks of treatment and at the 3-week follow-up, but no significant difference between the two groups was found [37].

Three trials used SNNA as the control intervention. One study reported a stronger effect of real acupuncture on VAS pain intensity in individuals with LBP after 8 weeks of treatment, but this difference was not significant at the 26- and 52-week follow-ups [33]. One study showed that knee OA patients reported an improved WOMAC pain index after 8 weeks of a real acupuncture intervention [17]. However, this difference declined over time and was no longer observed at the 52-week follow-up [17]. One study showed a greater reduction on the Brief Pain Inventory (BPI) by real acupuncture on chronic prostatitis after 10 weeks of treatment and at 24 weeks of follow-up [34].

Two trials used SNNA as an adjunct therapy to other interventions as the control intervention. One trial compared conventional orthopaedic therapy (COT) associated with real acupuncture or SNNA in treating LBP treatment [68]. In this study, real acupuncture plus COT showed better improvement in VAS pain intensity after 4 weeks of treatment and at the 3-month follow-up. The other study compared acupuncture or SNNA plus physiotherapy in treating LBP [32]. After 12 weeks of treatment and at the 9-month follow-up, there was no significant difference in VAS pain intensity between groups [32].

#### 3.4.11. Acupuncture versus Deep Needling at Nonacupoints

Twelve studies reported the effects of acupuncture versus deep needling at nonacupoints (DNNC). Fibromyalgia was evaluated by one study [41, 45, 46], shoulder [40, 42] and myofascial pain [38, 44] were assessed by two trials each, and headache [43], epicondylitis [47], LBP [48], pelvic pain [49], and neck pain [39] were evaluated by one study each. For fibromyalgia, two studies reported greater improvements in the VAS score in the real intervention group than in the DNNC group after 4 or 10 weeks of treatment and at the 1- and 2-, and 6- and 12-month follow-ups [41, 45], respectively. One study showed improvement in the NRS score after 18 sessions of real or sham acupuncture, but there was no significant difference between the groups [46].

For shoulder pain, 4 or 6 weeks of a real acupuncture intervention was superior to DNNC in reducing VAS pain intensity (an effect that lasted for up to 12 weeks) [40, 42]. For headaches, VAS pain intensity was significantly reduced after 5 weeks of a real acupuncture intervention and at the 3-month follow-up [43]. For epicondylitis, 5 weeks of real acupuncture treatment yielded improved VAS scores than needling at nonacupoints, but this difference was no longer observed at the 2-month and 1-year follow-ups [47]. Regarding myofascial pain, two trials showed improvements in pain intensity after the acupuncture treatment. One study showed VAS score improvement immediately after a single treatment [38], and the other study showed improvement after 8 sessions and at the 1-month follow-up [44]. Regarding chronic prostatitis, real acupuncture improved pelvic pain (CPSI) after 6 weeks of treatment and at the 8-, 16-, and 24-week follow-ups [49]. Neck pain showed similar results with a greater reduction in the VAS score in the acupuncture intervention group than in both the trigger point group and the nontrigger point group at the 6-week and at 12-week follow-ups [39]. For LBP, one study showed improvements in the CPGS score in the real intervention and DNNC groups, but no significant differences between them were found at the end of the treatment (5 to 7 weeks) or at the follow-up (24 weeks) [48].

#### 3.4.12. Acupuncture versus Nonpenetrating Needling

Eighteen studies reported the effects of real acupuncture versus nonpenetrating (fake) needles using the following approaches: blunt needle or needling simulation (with a toothpick) at real or nonacupoints. Three trials investigated knee OA [56, 59, 60], low back [51, 53, 64], or jaw myofascial pain [31, 54, 63]. Two studies investigated fibromyalgia [45, 61], headaches [52, 62], and shoulder pain [50, 58]. Arm [55], jaw [57], and neck [39] pain were investigated in one study each.

For LBP, a single real acupuncture treatment [53] or 4 weeks [64] of real acupuncture treatment improved VAS pain intensity compared to nonpenetrating needles at real or nonacupoints. However, this difference was no longer significant at the 12-week follow-up [64]. One study reported a neutral effect on the SBS score between real acupuncture and simulation after 7 weeks of treatment or at the follow-up at weeks 8, 26, and 52 [51]. For knee OA, one study showed using real acupuncture was more efficient in improving the WOMAC pain score after 8 weeks of treatment than using nonpenetrating needles at acupoints [60]. Two trials showed no difference between groups after 6 to 12 weeks of treatment and at the 26-week follow-up as measured by the WOMAC and KSS scores, respectively [56, 59]. Regarding jaw myofascial pain syndrome, three studies used single-day therapy. Two studies used nonpenetrating needles at real acupoints [31, 54], and one study used points away from real acupoints [63] as the control intervention. All three studies showed greater VAS score improvement in the real acupuncture group compared with the control group. Two studies evaluated fibromyalgia by using nonpenetrating needles at acupoints. In both studies, the VAS score was improved after 4 or 7 weeks of real treatment, and this effect remained significant after 1 to 3 months in the follow-up period, respectively [45, 61]. Two studies treated headaches by using nonpenetrating needles at acupoints and nonacupoints [52, 62]. After a brief treatment (one day) or 5 weeks of treatment, similar improvements in the VAS pain score were observed between the real and control groups [52, 62]. Notably, the pressure pain threshold increased for the real acupuncture group at the follow-up (6 weeks) [52]. Two studies treated shoulder pain by using nonpenetrating needles at acupoints [50] or trigger points [58]. Both studies showed improvement in the VAS and SPADI scores at the end of 5 or 6 weeks of treatment but not at the follow-up (20 weeks) [58]. Persistent arm pain was assessed by one study that used nonpenetrable needles at acupoints [55]. The authors showed that sham acupuncture was more effective than real acupuncture in reducing pain (VAS score) after 4 weeks of treatment, but this difference disappeared at the 1-month follow-up. Jaw pain and neck pain were studied by the same author [39, 57] who used using blunt needles at acupoints in the sham control group. The authors used a brief (single) acupuncture treatment as the intervention and showed a significant reduction in VAS pain intensity in both the treatment and control group immediately after the treatment and during the follow-up period (10 to 12 weeks in total).

#### 3.4.13. Acupuncture versus TENS Placebo

Four studies used inactive TENS as the control intervention for individuals with neck, shoulder, or low back pain. Two studies evaluated neck pain and reported greater VAS improvement in the acupuncture group than in the placebo group after 3 to 4 weeks of treatment [83, 84]. In addition, these effects remained stable after 6 to 12 months of the intervention. One study evaluated LBP and showed that acupuncture was more efficient than TENS placebo in reducing VAS pain intensity after 6 weeks of treatment, but this effect was no longer significant at the 6-month follow-up [86]. One study evaluated shoulder pain and showed significant effects of acupuncture compared with the control group on the NRS score after 3 weeks of treatment and at the 12-month follow-up [85].

#### 3.4.14. Acupuncture versus Laser or Sham Laser Acupuncture

One trial examined the effectiveness of acupuncture and a laser or sham laser at acupoints in treating chronic knee pain [87]. After twelve weeks of treatment, neither the laser nor needle acupuncture was superior to the sham laser and vice versa in reducing knee pain (NRS and WOMAC scores).

## 5. Discussion

In the present review, based on the data from RCTs, we evaluated the effectiveness of classical acupuncture (alone or as an adjunct therapy to other interventions) versus different types of control interventions in the treatment of chronic pain. We performed an extensive literature search and found 61 relevant RCTs. The proportion of high-quality studies reported here differs from those reported in previous systematic reviews that concluded the absence of [22] or the presence of very few [23] high-quality studies. The most common acupoints that were used in the selected studies were located along the meridians of the large intestine (LI), gallbladder (GB), stomach (ST), and spleen (SP), which are the most commonly used acupoints for a variety of painful conditions. Acupoints on the LI meridian (in particular LI-4) were used for almost all conditions (except for LBP and pelvis and hip pain), while GB acupoints were mainly used for neck, shoulder, back, and knee pain. Acupoints on the SP meridian were commonly used for pelvis and hip pain and fibromyalgia, and the ST acupoints (mainly ST-36) were commonly used for fibromyalgia, knee, and shoulder pain. LI-4, SP-6, GB-30 and 34, and ST-36 have long been used in clinical and experimental studies for pain and inflammation control [10, 91], and the stimulation of these points is related to the release of opioid peptides and the activation of brain areas involved in pain control [92, 93]. The largest benefits of real acupuncture were observed when it was compared to no treatment or waiting list control interventions (all 3 trials showed greater improvement with acupuncture), usual care (all 3 trials showed greater improvement), TENS placebo (all 4 trials showed greater improvement), and laser/sham laser acupuncture (all 2 trials showed greater improvement); in addition, large benefits were observed when acupuncture was combined with medical/usual care (all 5 trials showed greater improvement) and physiotherapy (all 3 trials showed greater improvement) compared to medical/usual care and physiotherapy alone. The limited benefits of real acupuncture were observed when it was compared to deep needling at nonacupoints (11 in 15 trials showed greater improvement), nonpenetrating needling (12 in 18 trials showed better improvement), and superficial needling (5 in 8 trials showed greater improvement). Real acupuncture was not superior to relaxation, physiotherapy, self-educational programs, or pharmacological interventions. The risk of adverse events related to acupuncture interventions appears to be low, but this issue was not reported or was vaguely reported by most of the studies.

Previous reviews showed that poor methodological quality designed studies tend to report positive results [23, 94–96]. This statement is in accordance with our data, in which all low-quality trials reported better pain outcomes in the true acupuncture group than in the control group, suggesting that some data from these studies may represent false positives. Considering all studies collectively, real acupuncture was superior to any nonpenetrating intervention, such as the TENS placebo and laser or sham laser treatments. Notably, most studies showed that real acupuncture was as effective as using nonpenetrating needles or superficial needling or deep needling at nonacupoints in reducing pain outcomes. Similar results were reported by other trials, which were scored as having high quality in the present review [32, 46, 55, 59], indicating an arbitrary effect of puncturing random points surrounding real acupoints. This effect may be partially explained by treatment expectation, as the expectation of needling can produce a larger placebo effect than acknowledging that no needling technique will be used [97, 98]. However, caution should be exercised when adopting this assumption, as most of the studies reviewed here did not implement good bias control, such as blinding patients, acupuncturists, and evaluators, reports of dropouts and withdrawals, and “Deqi” induction. These effects may be explained by the stimulation of local nerve fibers. Experimental and clinical studies have reported that needling nonacupoints in animals induces analgesia [99], and the anti-inflammatory effect of electroacupuncture is lost when nerve fibers supplying the stimulated area (dermatome) are damaged [10]. Superficial needling can stimulate nerve fibers within the dermatome of the real acupoint, and fake needles can stimulate nerve terminals underneath the acupoint, as these areas have higher electrical conductance than the surrounding tissue [100] and become hypersensitive under certain pathological conditions [101–103]. Therefore, the effects of sham acupuncture on pain control may replicate the effects of Japanese and Korean acupuncture [104]. In other words, sham acupuncture may not be appropriate as a control intervention for assessing the effectiveness of acupuncture.

Some points should be addressed as limiting factors in the present review. First, we included only articles written in the English language. Therefore, we cannot rule out that language selection can induce overbiased outcomes [105]. The second point is that we did not evaluate publication bias. Some specialized journals have the tendency to publish studies favoring a specific therapy. Third, we focused more on the quality rather than the quantity of the treatment sessions. The cumulative effect of acupuncture has been reported in clinical [106] and experimental [107] studies, and clinical trials have shown that brief acupuncture (removal of the needle immediately after reaching the desired depth or after Deqi feeling) immediately reduces pain [53, 62, 79, 80]. The last point is that some studies have reported that real acupuncture improved other scores not directly related to pain. For example, Miller et al. [56] showed no difference in the KSS pain score between the real and placebo acupuncture groups, but the real intervention showed better KSS knee and KSS function scores at the 12-week follow-up than the control intervention. The positives outcomes from the present review can be summarized as follows: (1) studies with poor methodological quality predict more positive outcomes; (2) acupuncture doses and the optimal technique should be examined in depth; (3) high-quality studies pointed that the perception of acupuncture treatment (real or fake) is sufficient to produce real analgesic effects; and (4) acupuncture (real or fake) is superior to conservative interventions (e.g., home exercises, physiotherapy, or TENS).

In general, our analysis demonstrated good evidence that receiving acupuncture is better than not receiving treatment or being put on a waiting list in terms of pain control. When it was compared to conventional or usual care, acupuncture presented slightly (reasonable) better outcomes. Limited evidence was found in placebo treatments that involve the expectation of needling (real or fake). However, conclusions about the effectiveness of acupuncture in treating chronic pain are still limited due to the low quality of the studies published.

## Figures and Tables

**Table 1:** List of criteria used to rank the methodological quality of the selected randomized controlled trials.

Selection bias
(i) Was the randomization method adequate?
(ii) Was the treatment allocation concealed?
(iii) Were all groups similar at baseline regarding the analyzed parameters?
Performance bias
(i) Was the practitioner background provided?
(ii) Was “Deqi” reported by the patient?
(iii) Was the patient blinded to the intervention?
(iv) Was the care provider blinded to the intervention?
(v) Was the compliance acceptable for all groups?
(vi) Was cointervention similar?
Detection bias
(i) Was the outcome assessor (evaluator) blinded to the intervention?
(ii) Was the timing of the outcome similar in all groups?
Attrition bias
(i) Were the dropouts and withdraws described?
(ii) Were all randomized patients analyzed inside their allocated group?
Results bias
(i) Was the data analysis fully described?
(ii) Was the study results free of suggestion or selective outcomes?
Other bias
(i) Was other potential bias source reported?

**Table 2:** Overall background for each painful condition.

Painful condition	Study (*n*)	Patients (total)	Patients (median)	Age (years)	Female (%)	Treatment sessions	Treatment length (weeks)	Acupoints used	Most common acupoints used
Arm	3	218	50 (47.5–86.5)	42.5 (38.7–47.5)	49.59 (48.795–55.395)	8 (8–8)	4 (4–4)	LI: 4, 5, 10, 11; LU: 5, 9; LV: 3; PC: 4–8; HT: 2, 7, 8; AP; EP: Sanjiao 5; TW: 5	LI: 4, 11

Low back	14	6736	137 (36.75–283.75)	49.8 (47–59.1)	59.04 (46.15–62)	9 (5.5–12)	6 (4–9)	UB: 20–34, 40, 50–54, 60, 62; GB: 30, 34; GV: 3–6; SP: 6; KI: 3; AP, EP: Huatuojiaji, Yaotongdian, Shiqizhuixia, and Extra 19	UB: 23, 25, 31, 32, 40, 60; GB: 30, 34; GV: 3, 4

Back neck	1	109	109	39	45	18	9	Most painful parts	

Fibromyalgia	5	433	75 (50–114)	46 (43.86–47)	100 (100–100)	9 (8–12)	8 (4–9)	LI: 4, 11; GB: 20, 34, 41; ST: 36, 44; SP: 6; LV: 3; UB: 10, 18, 20, 40, 62; HT: 7; PC: 6; GV: 14, 20; CV: 6; SI: 3, 4, 15; KI: 27	CV: 6; GB: 34; GV: 14, 20; HE: 7; LI: 4, 11; LV: 3; PC: 6; SP: 6; ST: 36

Headache	8	4296	102 (54.5–212.75)	43.655 (39.375–47.25)	82 (79–87.9)	13.5 (12–18.75)	12 (6–12)	GB: 8, 10, 14, 15, 20, 40–42; LI: 4; LV: 3, TE: 3, 5; GV: 16, 20, 24; UB: 2; SJ: 22, 23; EP: Extra 2, Taiyang	GB: 14, 20, 41; GV: 20; LI: 4; TE: 5; Taiyang

Pelvis and hip	3	221	89 (60.5–94.5)	40.9 (36.5–53.45)	37.5 (18.75–56.25)	6 (6–13)	6 (6–8)	GB: 29, 30, 34, 43; ST: 44; LI: 4; SP: 6, 9; UB: 33, 34, 54; CV: 1, 4; AP	CV: 1, 4; SP: 6, 9

Jaw	4	89	22.5 (17.5–27.25)	36.215 (32.0425–37.83)	82.825 (69.575–87.475)	3 (1–5.25)	4 (3.5–4.5)	LI: 4; ST: 6, 7; trigger points	LI: 4

Knee	6	1596	248 (189.25–291)	64.15 (63.1–68.8)	58.25 (52.055–65.525)	12 (12–16)	8 (8–12)	ST: 6, 34–36, 40, 43; SP: 4–6, 9, 10; UB: 11, 20–23, 39, 40, 57, 58, 60, 62; KI: 3, 10; GB: 30–36, 39, 41; LV: 3, 7–9; LI: 11; GV: 14, 20; EP: Xiyan	UB: 20, 57, 60; GB: 34, 36, 39; KI: 3, 10; LI: 11; LR: 3, 8; SP: 5, 6, 9, 10; ST: 34–36; Xiyan

Knee and hip	1	712	712	60.35	61	15	12	At the physicians' discretion	

Myofascial	3	114	39 (27–49.5)	45.2 (36.265–67.32)	93.75 (93.03–96.875)	8 (4.5–8.5)	4 (4–4)	LI: 4; GB: 20, 21; LV: 3, 20; AP	LI: 4

Neck	7	4659	123 (39.5–326)	47.3 (43.8–51.85)	69.9 (66.745–72.145)	8 (5.5–11)	4.5 (3.25–10.25)	GB: 8, 14, 20, 21, 34, 39, 41; LI: 4; LV: 3; UB: 2, 10, 17, 60, 64; LU: 7; TW:5; ST: 8, 36, 44; GV: 14, 20; SP: 6, 10; CV: 1–7: SI: 3, 11, 14; TE: 5, 15; AP, EP: Shiqizhui, Huatuojiaji Extra 1	GB: 20, 21, 34, 41; GV: 14; LI: 4; LV: 3

Shoulder	6	1206	147.5 (50–374.75)	53.54 (50.745–55.525)	55.155 (47.7925–69.755)	8 (5.25–13.75)	5 (4.25–5.75)	SI: 3, 9, 10, 11; LI: 4, 10, 11, 14–16; TE: 5, 13–15; GV: 14; ST: 36, 38; LU: 1, 2; GB: 21, 34; UB: 58; SJ: 14	GB: 21; LI: 4, 11, 14, 15; SI: 3, 9, 10, 11; ST: 36, 38; TE: 5, 14

SI: small intestine; ST: stomach; SP: spleen; KI: kidney; UB: urinary bladder; GB: gallbladder; LI: large intestine; LU: lung; LV: liver; PC: pericardium; HT: heart; CV: conception vessel; GV: governor vessel; AP: ashi acupoints; EP: extra acupoints; TW: triple warmer; TE: triple energizer.

**Table 3:** Quality score, number of studies, and efficiency of real acupuncture over the control group in each selected study.

Quality score	Number of studies	Acupuncture effect over the control group		
		Better	Worse	Same
7	1	1		
8	3	3		
9	4	2		2
10	7	5	1	1
11	6	5		1
12	16	13	1	2
13	12	11		1
14	9	6	1	2
15	1			1
16	2	1	1	

## Abbreviations

BPI: Brief Pain Inventory
CBNG: Cochrane Back and Neck Group
CNKI: China National Knowledge Infrastructure
COT: Conventional orthopaedic therapy
CP: Chronic pain
CPGS: Von Korff Chronic Pain Grade Scale
CPSI: Chronic Prostatitis Symptom Index
DNNC: Deep needling at nonacupoints
GB: Gallbladder
KSS: Knee Society Score
LBP: Low back pain
LI: Large intestine
MPQ: McGill Pain Questionnaire
NPQ: Northwick Park Questionnaire
NRS: Numeric rating scale
OA: Osteoarthritis
PRISMA: Preferred Reporting Items for Systematic Reviews and Meta-Analyses
RCTs: Randomized controlled trials
SBS: Symptom Bothersomeness Score
SES: Schmerz empfindung skala
SF-36: Short-Form Health Survey
SNA: Superficial needling at acupoints
SNNA: Superficial needling at nonacupoints
SP: Spleen
SPADI: Shoulder Pain and Disability
ST: Stomach
STRICTA: Standards for Reporting Interventions in Controlled Trials of Acupuncture
TCMLARS: Traditional Medical Literature Analysis and Retrieval System
TENS: Transcutaneous electrical nerve stimulation
VAS: Visual analogue scale
WOMAC: Western Ontario and McMaster Universities Osteoarthritis Index.

## Supplementary Materials

Supplementary Materials Search strategies on PubMed, EMBASE, and the Cochrane Central Register of Controlled Trials databases.
