# Supplementary material for: Classic Chinese Acupuncture versus Different Types of Control Groups for the Treatment of Chronic Pain: Review of Randomized Controlled Trials (2000–2018)
Source: Evid Based Complement Alternat Med. 2019 Dec 4;2019:6283912. doi: 10.1155/2019/6283912 (PMC6914898; doi:10.1155/2019/6283912)
Supplement: Supplementary Materials — Search strategies on PubMed, EMBASE, and the Cochrane Central Register of Controlled Trials databases. [file 6283912.f1.pdf]

Search strategies on Pubmed, Embase, and Cochrane Central Register of Controlled Trials databases.

Search Strategy in Pubmed

#1 Conditions: ((("chronic pain"[MeSH Terms])) OR (((((((((((((((((joint[Title/Abstract]) OR hand[Title/Abstract]) OR wrist[Title/Abstract]) OR shoulder[Title/Abstract]) OR back[Title/Abstract]) OR spine[Title/Abstract]) OR lumbar[Title/Abstract]) OR neck[Title/Abstract]) OR cervical[Title/Abstract]) OR hip[Title/Abstract]) OR knee[Title/Abstract]) OR arm[Title/Abstract]) OR leg[Title/Abstract]) OR limb[Title/Abstract]) OR jaw[Title/Abstract]) OR head[Title/Abstract]) OR pelvis [Title/Abstract])) OR (((((((((((((((((((((((((((((((((((((rotator cuff tendinitis) OR ankylosing spondylitis) OR fibromyalgia) OR carpal tunnel syndrome) OR joint disorders) OR osteoarthritis) OR arthritis) OR rheumatoid arthritis) OR elbow pain) OR back pain) OR neck pain) OR neck disorder) OR shoulder pain) OR knee pain) OR tennis elbow) OR lateral elbow tendinopathy) OR subacromial bursitis) OR lateral epicondylitis) OR myofascial pain) OR heel pain) OR epicondyalgia) OR ankle distorsions) OR rheumatic disorders) OR tendinitis) OR rotator cuff lesions) OR tempromandibular pain) OR tempromandibular dysfunction) OR hemiplegic shoulder) OR contracture) OR knee extensor mechanism disorders) OR myalgia) OR arthralgia) OR gonarthrosis) OR extremity) OR extremities)

#2 Acupuncture: ((((((acupuncture[MeSH Terms]) OR "acupuncture therapy"[MeSH Terms]) OR "acupuncture points"[MeSH Terms]) OR body acupuncture) OR triggers point)

#3 Study Design: (((((((((((((((random[Title/Abstract]) OR random[MeSH Subheading]) OR randomized controlled trial[Publication Type]) OR double blind method) OR single blind method) OR placebos) OR clinical trial[Publication Type]) OR clinical trials) OR controlled clinical trial[Publication Type])) OR

((clin*[Title/Abstract]) AND trial*[Title/Abstract])) OR ((((((singl*[Title/Abstract]) OR doubl*[Title/Abstract]) OR trebl*[Title/Abstract]) OR tripl*[Title/Abstract])) AND ((blind*[Title/Abstract]) OR mask*[Title/Abstract]))) OR placebo*[Title/Abstract]) OR random*[Title/Abstract])) OR random[MeSH Terms]

#4: #1 AND #2 AND #3 Filters: Publication date from 2000/01/01 to 2018/12/31

#5: ((review[Publication Type]) OR pilot[Publication Type]) OR protocol[Publication Type] Filters: Publication date from 2000/01/01 to 2018/12/31

#6: #4 NOT #5

Total :2120

Search Strategy in Embase

('acupuncture':ab,ti OR 'acupoint':ab,ti OR 'needle':ab,ti) AND ('chronic pain':ab,ti OR 'bone pain':ab,ti OR 'headache':ab,ti OR 'limb pain':ab,ti OR 'musculoskeletal pain':ab,ti OR 'myalgia':ab,ti OR 'neuralgia':ab,ti OR 'pelvic pain':ab,ti) AND [randomized controlled trial]/lim AND [article]/lim AND [humans]/lim AND [2000-2019]/py

Total :297

Search Strategy in Cochrane Central Register of Controlled Trials

ID Search Hits

#2 MeSH descriptor: [Chronic Pain] explode all trees 1663

#3 (joint):ti,ab,kw OR (hand):ti,ab,kw OR (wrist):ti,ab,kw OR (shoulder):ti,ab,kw OR (back):ti,ab,kw (Word variations have been searched) 69888

#4 (spine):ti,ab,kw OR (lumbar):ti,ab,kw OR (neck):ti,ab,kw OR (cervical):ti,ab,kw OR (hip):ti,ab,kw (Word variations have been searched) 54355

#5 (knee):ti,ab,kw OR (arm):ti,ab,kw OR (leg):ti,ab,kw OR (limb):ti,ab,kw OR (jaw):ti,ab,kw (Word variations have been searched) 118117

#6 (head):ti,ab,kw OR (pelvis):ti,ab,kw (Word variations have been searched) 22352

#7 MeSH descriptor: [Acupuncture] explode all trees 140

#8 MeSH descriptor: [Acupuncture Therapy] explode all trees 4184

#9 MeSH descriptor: [Acupuncture Points] explode all trees 1764

#10 MeSH descriptor: [Randomized Controlled Trial] explode all trees 138

#11 (randomized controlled trial):pt OR (clinical trial):pt OR (controlled clinical trial):pt OR (double blind):pt OR (random):ti,ab,kw (Word variations have been searched) 901390

#12 #2 or #3 or #4 or #5 or #6 216860

#13 #7 or #8 or #9 4273

#14 #10 or #11 901390

#15 #12 and #13 and #14 with Cochrane Library publication date Between Jan 2000 and Dec 2018, in Trials 1318
